# Leucine‐Rich Repeat Containing 15 Promotes the Inflammatory Response in Rheumatoid Arthritis by Regulating NF‐κB Pathway

**DOI:** 10.1002/iid3.70220

**Published:** 2025-07-11

**Authors:** Miaomiao Xin, Guangtao Xia, Xin Guan, Guangmin Xi, Min Fu

**Affiliations:** ^1^ Department of Rheumatology and Immunology Shandong Provincial Hospital Affiliated to Shandong First Medical University (Shandong Provincial Hospital) Jinan Shandong China; ^2^ College of Life Sciences Qilu Normal University Jinan Shandong China

**Keywords:** fibroblast‐like synoviocytes, inflammatory response, Lrrc15, NF‐κB pathway, rheumatoid arthritis

## Abstract

**Purpose:**

To explore the influence and molecular mechanism of leucine‐rich repeat containing 15 (LRRC15) in rheumatoid arthritis (RA) model induced by collagen‐induced arthritis (CIA) in rats and interleukin‐1 beta (IL‐1β) treated fibroblast‐like synoviocytes (FLSs).

**Methods:**

LRRC15 expression was analyzed using reverse transcription quantitative polymerase chain reaction (RT‐qPCR), western blot analysis, and immunohistochemistry. Hematoxylin‐eosin (H&E) and safranin‐O‐green staining were performed to assess the pathological changes in the joint tissues of rats. The messenger ribonucleic acid (mRNA) and protein expression of interferon gamma (IFN‐γ), interleukin (IL)‐6, IL‐1β, and IL‐10 was detected by RT‐qPCR, enzyme‐linked immunosorbent assay (ELISA), and western blot. Cell proliferation and migration was surveyed using cell counting kit‐8 (CCK‐8), 5‐Ethynyl‐2′‐deoxyuridine (EdU), and Transwell assays. Western blot was applied to detect the protein changes of nuclear factor kappaB (NF‐κB) subunit p65, phosphorylated (p)‐p65, inhibitor of kappa Bα (IκBα), phosphorylated inhibitor kappa Bα (p‐IκBα), and nuclear factor erythroid 2‐related factor 2 (Nrf2).

**Results:**

In CIA rats and IL‐1β‐treated FLSs, LRRC15 expression was upregulated. In vivo, *Lrrc15* silencing prevented joint damage, inhibited the expression of IFN‐γ, IL‐6, IL‐1β, and p65, and increased the expression of IL‐10 and IκBα. In vitro, *Lrrc15* silencing inhibited the proliferation, migration, and the expression of IFN‐γ, IL‐6, IL‐1β, p‐p65, and p‐IκBα, and increased the expression of IL‐10, IκBα, and Nrf2 in FLSs.

**Conclusion:**

*Lrrc15* silencing relieved joint damage and inflammatory response in RA, and this may be associated with the inhibition of the NF‐κB pathway.

## Graphical abstract

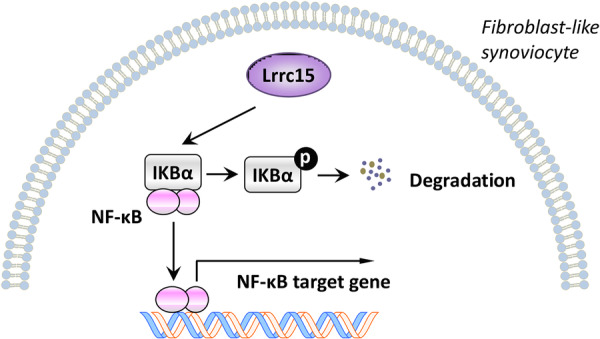

**Abbreviations**
CIA: collagen‐induced arthritis
CIA: collagen‐induced arthritis
ECM: cell‐cell and cell‐extracellular matrix
ELISA: enzyme‐linked immunosorbent assay
FLSs: fibroblast‐like synoviocytes
IL‐1β: interleukin‐1 beta
IL‐1β: interleukin‐1 beta
KEGG: Kyoto Encyclopedia of Genes and Genomes
Lrrc15: leucine‐rich repeat containing 15
RA: rheumatoid arthritis
RT‐qPCR: reverse transcription quantitative polymerase chain reaction

## 1 Introduction

Rheumatoid arthritis (RA) is a systemic autoimmune disease characterized by joint destruction, pain, hyperplasia of fibroblast‐like synoviocytes (FLSs), and chronic inflammation [1, 2, 3]. Stiffness or deformity of the joint may occur in the late stages of RA, resulting in serious impairment of joint function [4]. Although the majority of patients experience remission through the application of biological agents and disease‐modifying anti‐rheumatic drugs, some do not respond to current treatments [5, 6]. For instance, nonsteroidal anti‐inflammatory drugs (NSAIDs) for inhibition of COX‐2 will lead to bleeding and gastrointestinal ulceration [7]. Another anti‐inflammatory drug, glucocorticoids, will result in side effects including nausea, abdominal pain, ulcers, osteoporosis, and diabetes [8]. Therefore, understanding the pathogenesis of these mechanisms and developing new therapeutic targets are important approaches for the treatment of RA.

As a transmembrane protein, leucine‐rich repeat containing 15 (LRRC15) is located on chromosome 3 at 3q29 [9]. *Lrrc15* has been reported to be participates in the interactions of cell–cell and cell–extracellular matrix (ECM) [9]. In various tumor types such as ovarian, pancreatic, and breast cancer, *Lrrc15* expression is upregulated [10, 11, 12]. Satoh et al. identified a novel rat gene named *Lib*, and the human counterpart is located on chromosome 3q29 [13]. Moreover, in rat C6 astrocytoma cells, *Lib* was observed to be upregulated in response to pro‐inflammatory cytokines, including tumor necrosis factor‐α (TNF‐α), interleukin‐1 beta (IL‐1β), and interferon gamma (IFN‐γ). Notably, *Lrrc15* was observed to be upregulated in caries‐diseased pulpal tissue [14]. Wang et al. revealed that *Lrrc15* plays a regulatory role in the osteogenic differentiation of mesenchymal stem cells [15]. However, little is known regarding the function of *Lrrc15* in the context of RA.

In the present study, we established an RA rat model induced by the collagen‐induced arthritis (CIA) and inflammatory cell model induced by IL‐1β to explore the effects and underlying mechanism of *Lrrc15* in RA. Our study provides a theoretical basis for the development of novel RA therapies.

## 2 Materials and Methods

### 2.1 Single‐Cell Sequencing

The GSE243917 data set [16] was used for single‐cell sequencing. This data set comprises samples collected from 11 patients with RA, which were obtained through single‐cell RNA sequencing technology. The data preprocessing steps encompass the following aspects. Initially, the data is filtered based on quality control metrics for cells, such as the proportion of mitochondrial gene expression (set at ≤ 20%), unique molecular identifiers (UMIs), and gene counts (ranging from 100 to 150,000 and 200 to 10,000), to ensure the quality of the data. After the screening process, we obtained transcriptomic data for 21,968 cells. The scRNA‐seq data were normalized using the “NormalizeData” function and scaled using the “ScaleData” function. The top 2000 highly variable genes were identified using the “FindVariableFeatures” function. Subsequently, we employed the “RunPCA” function to reduce the dimensionality of the scRNA‐seq data. To integrate single‐cell data from different samples for unsupervised clustering, the “RunHarmony” function from Harmony was utilized [17]. The selection of principal components (PCs) was conducted by ranking them using the ElbowPlot function from the Seurat package. The first 30 principal components (PCs) were employed for Uniform Manifold Approximation and Projection (UMAP) analysis. Subsequently, a single‐cell map with a resolution of 0.1 was generated using the “FindClusters” function. In addition, the “FindAllMarkers” function was utilized to detect gene expression markers. Following this, we annotated the cell types in the study using cell marker genes. The potential pathways involved in *Lrrc15* were analyzed using the Kyoto Encyclopedia of Genes and Genomes (KEGG).

### 2.2 Animals

Male Wistar rats (5 ~ 6 weeks) were purchased from Jinan Pengyue Experimental Animal Breeding Co. Ltd. (China). In specific pathogen‐free conditions under a humidity of 55% ± 5% at 22°C ± 2°C, rats were provided free access to diet and water. All experiments were approved by the Animal Care and Use Committee of Shandong Provincial Hospital Affiliated to Shandong First Medical University (Shandong Provincial Hospital).

### 2.3 Grouping and CIA Animal Model

The rats were divided into four groups (*n* = 6), including control (untreated), CIA (CIA induction), CIA + Ad‐scramble (CIA induction and injection with adenovirus control [Ad‐scramble]), and CIA + Ad‐shLrrc15 (CIA induction and injection with Ad‐shLrrc15). For CIA induction, bovine type II collagen (Sigma, USA) was first dissolved in 0.05 M acetic acid and then emulsified in complete Freund's adjuvant (Sigma). Next, rats were administered 200 μL emulsions into the base of the tail by intradermal injection. After the first injection for 7 days, rats received a booster vaccination with 200 μL emulsions in which incomplete Freund's adjuvant was used to emulsify bovine type II collagen. After the first injection, in the CIA + Ad‐scramble and CIA + Ad‐shLrrc15 groups, Ad‐scramble and Ad‐shLrrc15 (1 × 10^10 ^pfu) were injected into the joint cavity of rats every 5 days from Day 5 after the first immunization to Day 15. All rats were weighed every 4 days, and arthritis was scored every 2 days. Forty days after the first injection, the rats were killed, and joint tissues were obtained for subsequent experimental analysis.

### 2.4 Isolation and Culture of Primary FLS

FLSs were isolated from the synovial tissues of normal Wistar rats. Briefly, synovial tissues were cut into 1–2 mm^3^ pieces and digested with DMEM (Gibco, USA) containing collagenase IV (1 mg/mL) for 1 h at 37°C. After filtering and centrifugation at 400*g* for 5 min, FLSs were cultured in DMEM containing 10% fetal bovine serum (FBS; Gibco) at 37°C in 5% CO_2_. FLSs from passages 2–6 were used for subsequent experiments.

### 2.5 Cell Transfection and Treatment

Guangzhou RiBoBio Biotechnology Co. Ltd. (China) provided *Lrrc15* small interfering RNA (siRNA) (siLrrc15; 5′‐GCTGAAACATTACCTCCTCTT‐3′) and scrambled siRNA (siNC; 5′‐GTTCCCCCATACTACTTAGAT‐3′). Based on the manufacturer's instructions, siLrrc15 and siNC were transfected into FLSs using Lipofectamine 2000 (Invitrogen). After transfection for 48 h, FLSs in siNC and siLrrc15 groups were treated with 10 ng/mL IL‐1β for 24 h, and FLSs were then collected for subsequent experiment.

### 2.6 Immunohistochemical Staining

The synovial tissues of rats were fixed with 4% paraformaldehyde, embedded in paraffin, and cut into 5 μm‐thick sections. Sections were then treated with xylene (Sigma) and rehydrated in gradient ethanol (Sigma). To inhibit endogenous peroxidase activity, 3% hydrogen peroxide was used to treat sections for 15 min at room temperature. To recover the antigen, 0.01 M citrate buffer was applied to treat sections for 20 min at 80°C. Subsequently, sections were blocked with normal goat serum for 30 min at room temperature and then incubated with anti‐Lrrc15 antibody (1:50; #50546, Cell Signaling Technology, USA) at 4°C overnight, and this was followed by incubation with secondary antibody (1:100; ab7090, Abcam, USA). Finally, sections were stained using 3,3′‐diaminobenzidine (Sigma) and then observed under a microscope (Olympus, Japan).

### 2.7 H&E Staining

After dewaxing and rehydration, sections of the rat joint tissues were stained with hematoxylin (Solarbio, China) for 5 min and treated with eosin for another 1 min. Finally, histological changes in the rat joint tissues were observed under a microscope, and arthritis scores were evaluated in a blinded manner by two investigators. The scoring criteria were as follows. (A) Inflammatory cell infiltration: 0, none; 1, mild; 2, moderate; 3, severe. (B) Hyperplasia was defined as synovial tissue intimately invading the bone and/or cartilage and scored from 0–3 as follows: 0, none; 1, minimal; 2, moderate; 3, severe.

### 2.8 Safranin‐O‐Fast Green Staining

After dewaxing and hydration, sections of rat joint tissues were stained with Fast Green Staining solution for 5 min and washed with running water. The sections were exposed to 0.5% hydrochloric acid alcohol for 10 s, and then stained with 0.1% safranin O solution (Solarbio) for 5 min. Subsequently, the sections were dehydrated and mounted with neutral balsam. Finally, the sections were observed under a microscope and scored using OARSI. Briefly, the OARSI was scored from 0–6 according to the following criteria: 0, surface intact and cartilage intact; 1, surface intact; 2, surface discontinuity; 3, vertical fissures; 4, erosion; 5, denudation; 6, deformation.

### 2.9 Enzyme‐Linked Immunosorbent Assay (ELISA)

After the specified treatment, FLSs were centrifuged at 1000*g* for 5 min to obtain the supernatant. The levels of inflammatory factors were analyzed using IFN‐γ (E‐EL‐R0009), IL‐6 (E‐EL‐R0015), IL‐1β (E‐EL‐R0012), and IL‐10 (E‐EL‐R0016) ELISA kits (Elabscience, Wuhan, China) according to the manufacturer's instructions.

### 2.10 Reverse Transcription Quantitative Polymerase Chain Reaction (RT‐qPCR)

Total RNA was isolated from FLSs and rat synovial tissues using TRIzol reagent (Invitrogen). Next, cDNA was synthesized using the RevertAid First Strand cDNA Synthesis Kit (Thermo Fisher Scientific, USA). In line with the instructions for the Power SYBR Green PCR Master Mix (Roche, Switzerland), RT‐qPCR was performed on a 7500 Real‐Time PCR Detection System (Applied Biosystems, USA). The relative mRNA expression was counted through the $2^{‐\Delta \Delta Ct}$ method. Shanghai Sangon Biotech Co. Ltd. (Shanghai, China) synthesized the primers, and the primer sequences were as follows: *Lrrc15* (F: 5′‐TGGGGTGCACTTCACAAACT‐3′, R: 5′‐ GGGATCTGGAGGGGAAGTCT‐3′), *Ifng* (F: 5′‐ TGTTACTGCCAAGGCACACT‐3′, R: 5′‐TGTGGGTTGTTCACCTCGAA‐3′), *Il6* (F: 5′‐AGAGACTTCCAGCCAGTTGC‐3′, R: 5′‐TGCCATTGCACAACTCTTTTC ‐3′), *Il1b* (F: 5′‐ CAGCTTTCGACAGTGAGGAGA ‐3′, R: 5′‐ CTCCACGGGCAAGACATAGG ‐3′), *Il10* (F: 5′‐ TTCCCTGGGAGAGAAGCTGA ‐3′, R: 5′‐ GACACCTTTGTCTTGGAGCTTA ‐3′), *Nfkb* (p65) (F: 5′‐ CGATGCATCCACAGCTTCCAG ‐3′, R: 5′‐ TAATGGCTTGCTCCAGGTCTC ‐3′), *Ikba* (F: 5′‐ TCACGGAAGATGAGTTGCCC ‐3′, R: 5′‐ CAAGTCCACGTTCCTTTGGC ‐3′), and *Gapdh* (internal control; F: 5′‐ CTCTCTGCTCCTCCCTGTTC ‐3′, R: 5′‐ CGATACGGCCAAATCCGTTC‐3′).

### 2.11 Western Blot

FLSs were treated by RIPA lysis buffer (Beyotime Biotechnology) to extract proteins. Next, 30 μg proteins were loaded onto SDS‐PAGE and transferred to PVDF membranes. The membrane was blocked using 5% skim milk for 2 h at room temperature and then incubated with anti‐LRRC15 (1:1000; ab150376, Abcam, USA), anti‐IFN‐γ (1:1000; ab267369, Abcam), anti‐IL‐6 (1:1000; ab233551, Abcam), anti‐IL‐10 (1:2000; 60269‐1‐Ig, Proteintech), anti‐p65 (1:1000; #8242, Cell Signaling Technology), anti‐phosphorylated (p)‐p65 (1:1000; #3033, Cell Signaling Technology), anti‐p‐IKBα (1:1000; #2859, Cell Signaling Technology), anti‐IKBα (1:1000; #4812, Cell Signaling Technology), anti‐Nrf2 (1:1000; #20733, Cell Signaling Technology), and anti‐GAPDH (1:5000; 10494‐1‐AP, Proteintech) overnight at 4°C. This was followed by incubating with anti‐rabbit (1:2000; #7074, Cell Signaling Technology) or anti‐mouse (1:2000; #7076, Cell Signaling Technology) secondary antibodies for 1 h at room temperature. Finally, protein bands were visualized using an enhanced chemiluminescence kit (Beyotime Biotechnology). For protein quantification, Image J software (USA) was used.

### 2.12 Cell Counting Kit‐8 (CCK‐8) Assay

After specified treatments for 0, 12, 24, 48, and 72 h, FLSs were treated with 10 μL of CCK‐8 reagent (Beyotime Biotechnology) and cultured for 2 h at 37°C. Finally, the optical density (OD) was measured at 450 nm using a microplate reader (Bio‐Rad).

### 2.13 5‐Ethynyl‐2′‐Deoxyuridine (EdU) Staining

After specified treatment, FLSs were exposed to 10 μM of EdU (Beyotime Biotechnology Co. Ltd.) for 4 h at 37°C. Next, at room temperature, FLSs were fixed with 4% formaldehyde (Sigma) for 20 min. Following that, FLSs were incubated with 0.5% Triton X‐100 for 10 min, treated with ×1 Apollo reaction cocktail for 30 min, and stained with DAPI for 30 min. EdU‐positive cells were observed and counted under a fluorescence microscope (Olympus, Tokyo, Japan).

### 2.14 Transwell Migration Assay

The lower chamber of transwell was filled with 600 μL of complete medium containing 20% fetal bovine serum (FBS, Sigma). After the specified treatment, FLSs were suspended in serum‐free medium in the upper chamber of a Transwell plate (Corning, USA). After incubation at 37°C for 48 h, migrated FLSs were fixed with 4% paraformaldehyde for 20 min at room temperature. Then, FLSs were stained with 0.1% crystal violet (Sigma) for 10 min, and the migrated FLSs were imaged and counted under an inverted optical microscope (Olympus).

### 2.15 Immunofluorescence Staining

After specified treatment at room temperature, FLSs were fixed with 4% paraformaldehyde for 15 min and then permeabilized with 0.5% Triton X‐100 for 15 min. After blocking with 5% BSA for 1 h, FLSs were immunostained with anti‐ NF‐κB (p65) primary antibody (1:500; #8242, Cell Signaling Technology) overnight at 4°C, and then incubated with a secondary antibody (1:1000; #4412, Cell Signaling Technology) for 1 h at room temperature. DAPI was used to counterstain the nuclei for 10 min. The stained cells were observed under a fluorescence microscope.

### 2.16 Statistical Analysis

GraphPad Prism software (USA) was utilized to carry out statistical analysis. Data are presented as mean ± standard deviation (SD). Statistical difference between the two groups was analyzed using unpaired *t*‐test. Statistical difference between three or more groups was performed by one‐way ANOVA followed by Tukey's post hoc test or two‐way ANOVA followed by Bonferroni's post hoc test. When *p* value was less than 0.05, statistical significance was accepted.

## 3 Results

### 3.1 LRRC15 Is Highly Expressed in the Synovial Tissues of CIA Rats

**Figure 1:**
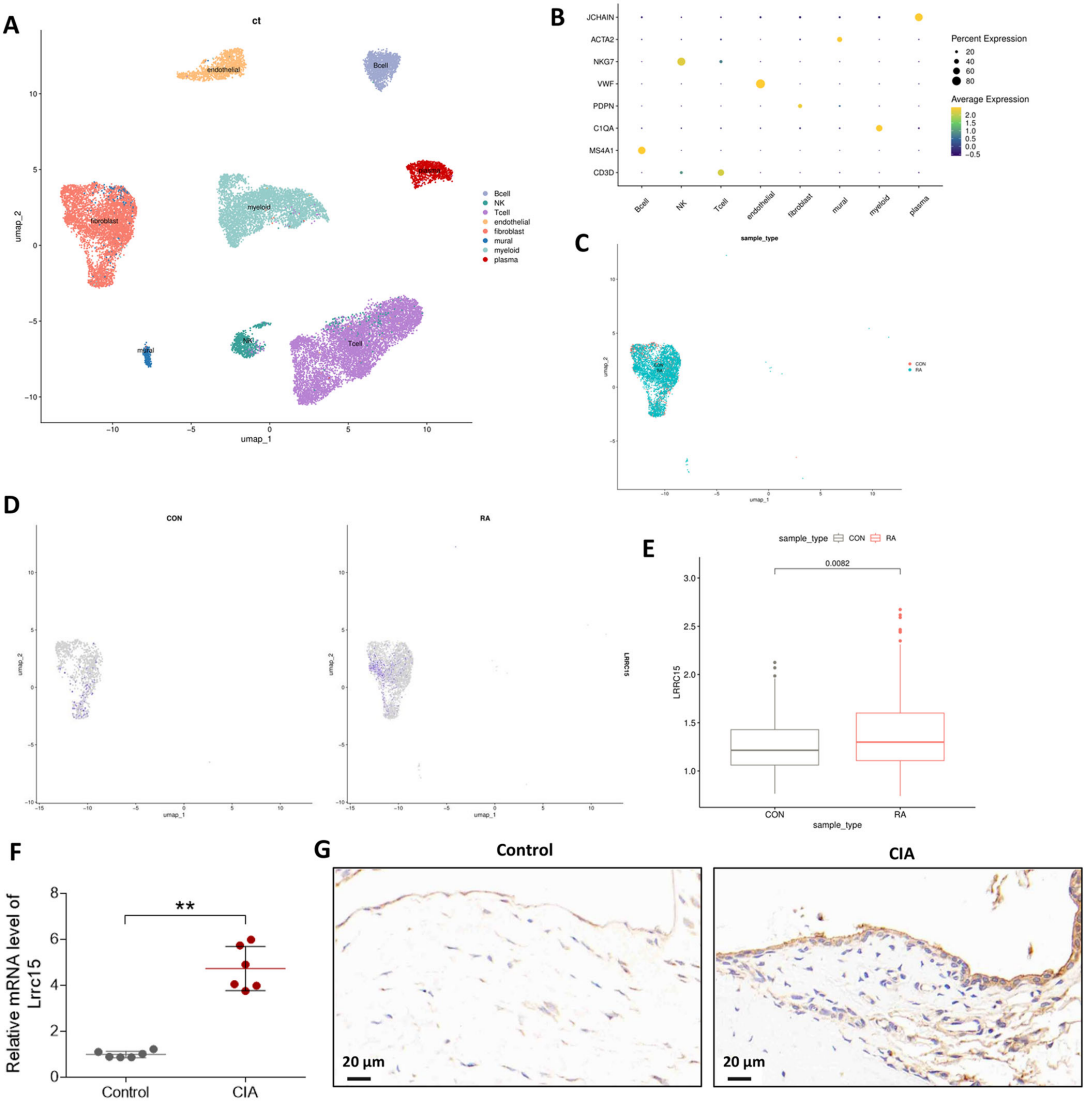
Expression of LRRC15 in the synovial tissues of CIA rats. (A) Cell atlas of RA tissues. (B) Cell markers for marking different cell types in cell clusters. (C) The UMAP plot of fibroblasts in RA tissues. (D and E) The expression of *Lrrc15* in the fibroblasts of control and RA tissues. (F and G) Wistar rats were injected with bovine Collagen II emulsified in complete Freund's adjuvant for the establishment of CIA animal models. The expression of LRRC15 in the synovial tissues of control and CIA rats was detected by RT‐qPCR (F) and immunohistochemistry (G). Data were presented as mean ± SD and analyzed using the unpaired *t*‐test. *N* = 6. **p < 0.01.

To uncover the key pathomechanisms in RA, we performed single‐cell sequencing to display the different cell populations that exist in RA (Figure 1A). Different cell markers were used to label different cell types in the cell populations (Figure 1B). It was shown that RA tissues have a large population of fibroblasts and LRRC15 was highly expressed in fibroblasts of the RA group as compared to those in control group (Figure 1C–E).

Next, we constructed a CIA rat model (an animal model of RA) and detected *Lrrc15* expression in synovial tissues using RT‐qPCR. As presented in Figure 1F, *Lrrc15* was significantly overexpressed in the CIA group compared to Lrrc15 levels in the control group. Additionally, immunohistochemistry confirmed that the LRRC15 protein levels were higher in the synovial tissues of CIA rats than they were in those of control rats (Figure 1G).

### 3.2 Silencing of *Lrrc15* Prevents Joint Damage in CIA Rats

**Figure 2:**
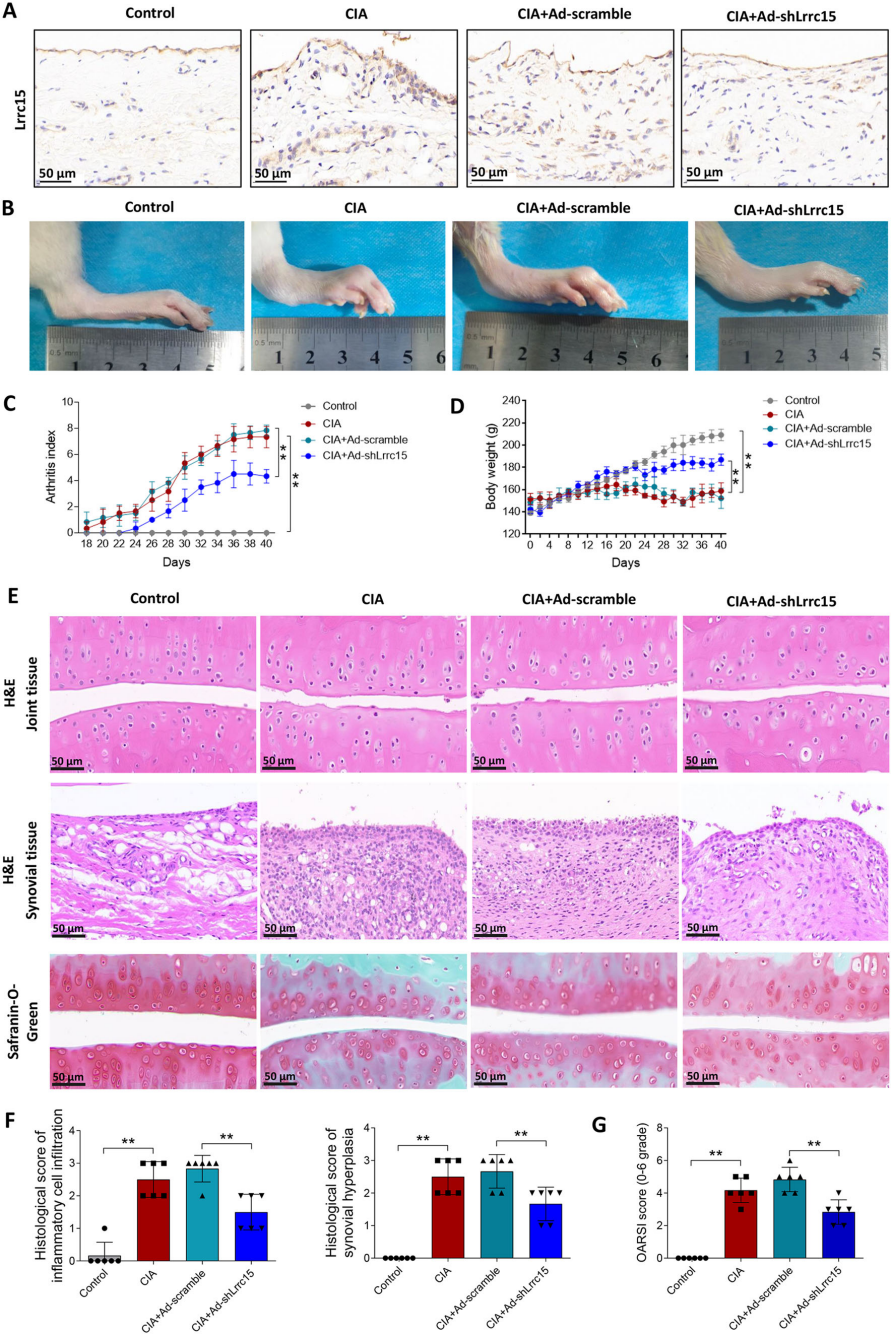
Functional effects of *Lrrc15* on joint damage in CIA rats. Ad‐scramble or Ad‐shLrrc15 was injected into the both joint cavity of CIA rats. CIA and control group of rats injected with same volume of vehicle. (A) LRRC15 protein levels in the synovial tissues of rats were detected using immunohistochemistry. (B) Macroscopic evidence of arthritis, such as erythema or swelling, in the hind paw of each group of rats. (C) The arthritis index in rat was evaluated every 2 days. (D) Body weight of rat was weighed every 4 days. (E) Representative images of H&E staining in joint and synovial tissues and Safranin‐O‐Green staining in joint tissues. (F) The pathological score of inflammatory cell infiltration and synovial hyperplasia in synovial tissues of rat. (G) The OARSI score in joint tissues of rat. Data were presented as Mean ± SD and analyzed using the one‐way ANOVA. *N* = 6. ** *p* < 0.01.

To investigate the function of *Lrrc15* in RA, we injected Ad‐shLrrc15 into CIA rats. Immunohistochemistry staining images confirmed the decrease of LRRC15 expression in rats injected with Ad‐shLrrc15, as compared to the rats injected with Ad‐scramble (Figure 2A). As presented in Figure 2B,C, CIA rats developed a severe form of arthritis, as seen by the remarkable increases in soft tissue swelling and the arthritis index. Silencing of *Lrrc15* reduced the soft tissue swelling and arthritis index compared with CIA + Ad‐scramble rats. Figure 2D indicates that the body weight of the control group exhibited a steady growth trend. After modeling for 20 days, the body weights of CIA and CIA + Ad‐scramble rats stopped growing, while silencing of *Lrrc15* alleviated weight loss in rats. Next, the joint and synovial tissues of the rats were stained with H&E. Figure 2E,F indicate that the joint tissues of CIA and CIA + Ad‐scramble rats exhibited significant cartilage damage, inflammatory cell infiltration, and synovial hyperplasia, but silencing of *Lrrc15* alleviated those symptoms. The results of Safranin‐O‐Green staining suggested that the joint tissues of CIA and CIA + Ad‐scramble rats presented significant cartilage matrix degradation and higher OARSI score, while silencing of *Lrrc15* alleviated cartilage matrix degradation and reduced the OARSI score (Figure 2E,G).

### 3.3 Silencing of *Lrrc15* Attenuates Pro‐Inflammatory Cytokine Levels in CIA Rats

**Figure 3:**
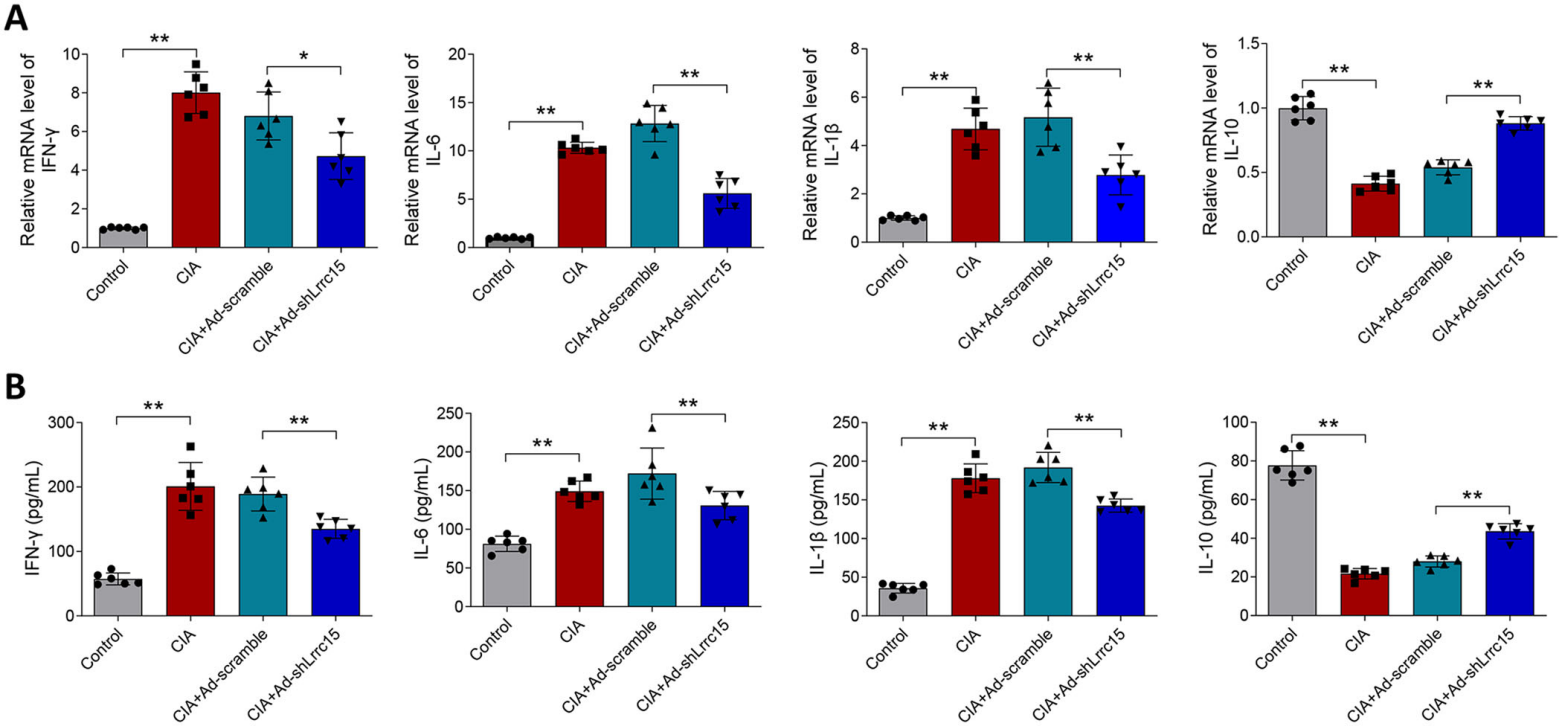
Functional effects of *Lrrc15* on pro‐inflammatory cytokine levels in CIA rats. Ad‐scramble or Ad‐shLrrc15 was injected into both joint cavities of CIA rats. CIA and control group of rats injected with same volume of vehicle. (A) The mRNA changes of *Ifng*, *Il6*, *Il1b*, and *Il10* in synovial tissues of rats were examined using RT‐qPCR. (B) The levels of IFN‐γ, IL‐6, IL‐1β, and IL‐10 in synovial fluid of rats were assessed using ELISA. Data were presented as Mean ± SD and analyzed using the one‐way ANOVA. *N* = 6. *p < 0.05; **p < 0.01.

Subsequently, joint inflammatory changes in rats were examined using RT‐qPCR. As presented in Figure 3A, after CIA induction, the mRNA levels of pro‐inflammatory cytokines *Ifng*, *Il6*, and *Il1b*, were increased, but the mRNA level of anti‐inflammatory cytokine *Il10* was decreased. However, silencing of *Lrrc15* reduced the mRNA levels of *Ifng*, *Il6*, and *Il1b* and enhanced *Il10* mRNA levels. Additionally, the changes of IFN‐γ, IL‐6, IL‐1β, and IL‐10 in the synovial fluid of rats were examined using ELISA. Figure 3B indicates that the levels of the pro‐inflammatory cytokines IFN‐γ, IL‐6, and IL‐1β in the CIA and CIA + Ad‐scramble groups were higher than that in the control group, but the IL‐10 level was lower than that in the control group. Compared to CIA + Ad‐scramble, silencing of *Lrrc15* alleviated the increase in levels of IFN‐γ, IL‐6, and IL‐1β and the decrease of IL‐10 levels.

### 3.4 Silencing of *Lrrc15* Induces the Inhibition of NF‐κB Signaling Pathway in CIA Rats

**Figure 4:**
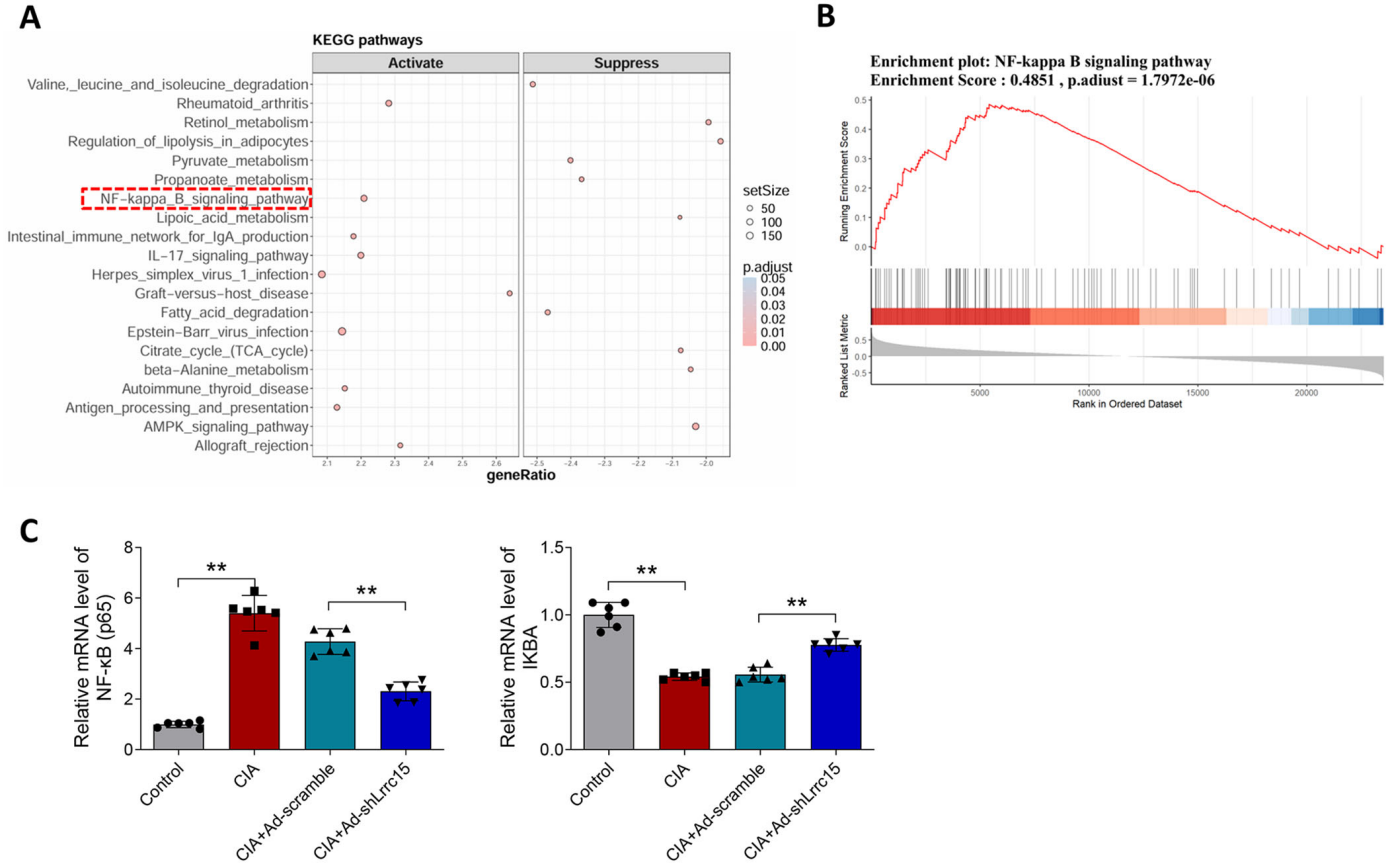
Regulation of *Lrrc15* on the NF‐κB signaling pathway in CIA rats. (A) KEGG analysis was performed to seek the pathways that were regulated by *Lrrc15*. (B) Enrichment score of the NF‐κB signaling pathway that can be positively regulated by *Lrrc15*. (C) The key factors of the NF‐κB signaling pathway, *Nfkb* (p65) and *Ikba*, were detected using RT‐qPCR in each group of rats. Data were presented as Mean ± SD and analyzed using the one‐way ANOVA. *N* = 6. **p < 0.01.

KEGG pathway enrichment analyses indicated the factors that can be regulated by *Lrrc15*, including the NF‐κB signing pathway, the IL‐17 signaling pathway, and others (Figure 4A). Figure 4B indicates that *Lrrc15* could activate the NF‐κB signaling pathway. Next, the key factors of the NF‐κB signaling pathway, *Nfkb* subunit (p65) and *Ikba*, were detected using RT‐qPCR. Figure 4C indicates that the CIA and CIA + Ad‐scramble groups exhibited higher mRNA expression of *Nfkb* (p65) and lower mRNA expression of *Ikba* in the joint tissue of rats, while silencing of *Lrrc15* decreased the *Nfkb* (p65) mRNA expression and increased the *Ikba* mRNA expression.

### 3.5 Silencing of *Lrrc15* Attenuates IL‐1β‐Induced FLS Activation

**Figure 5:**
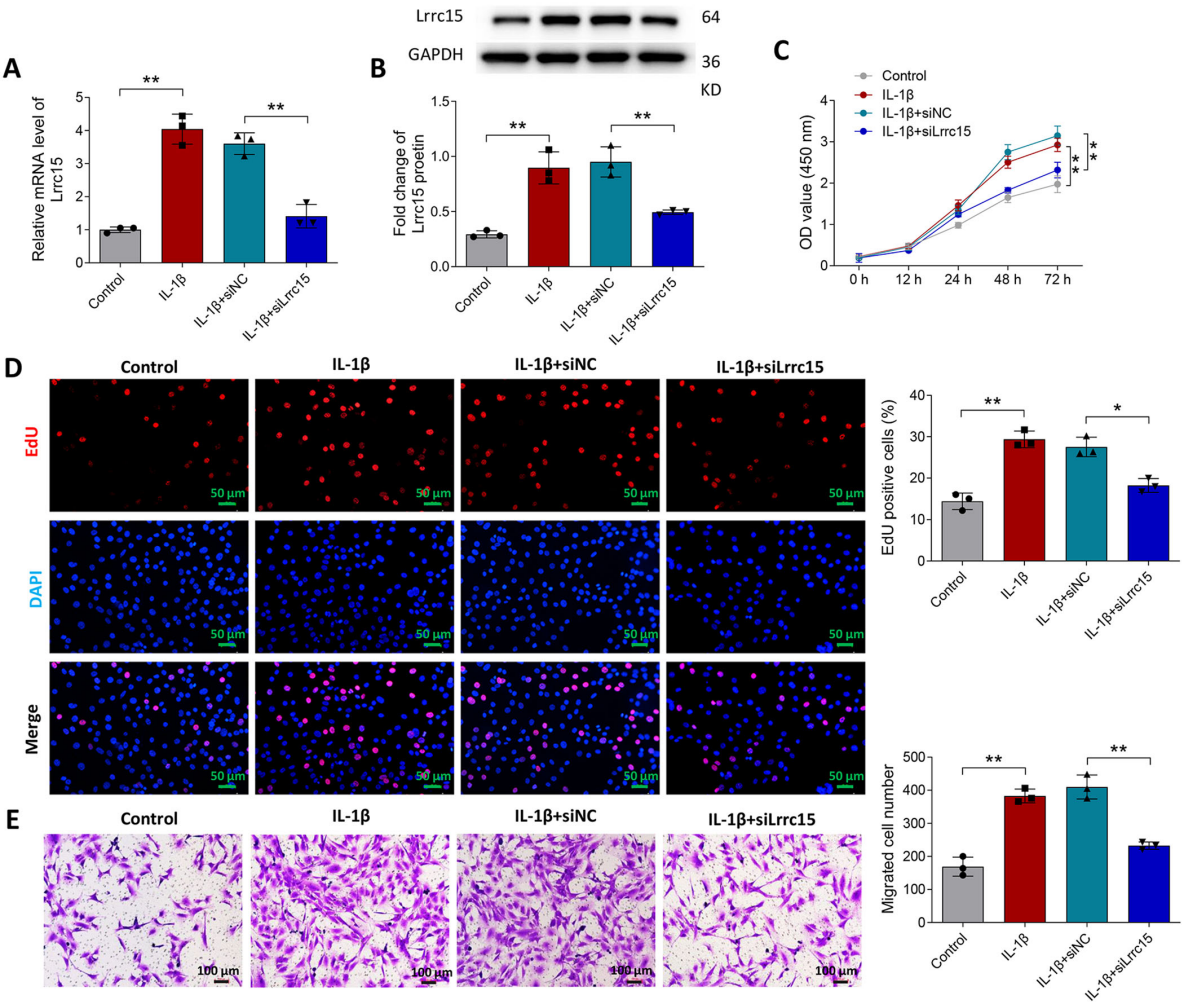
Effects of *Lrrc15* on IL‐1β‐induced FLS activation in vitro. (A–E) Primary rat FLSs transfected with siNC or siLrrc15 were treated with 10 ng/mL IL‐1β for 24 h. LRRC15 expression was measured by RT‐qPCR (A) and western blot (B). The proliferation of FLSs was detected by CCK‐8 (C) and EdU staining (D). The migration of FLSs was assessed by Transwell assay (E). Data were presented as Mean ± SD and analyzed using the one‐way or two‐way ANOVA. *N* = 3. *p < 0.05; **p < 0.01.

To further probe into the effects of *Lrrc15* on RA, FLSs were stimulated with IL‐1β to construct a RA cell model and transfected with si‐Lrrc15 or si‐NC. As presented in Figure 5A,B, IL‐1β increased LRRC15 expression, while si‐Lrrc15 transfection significantly inhibited LRRC15 expression in FLS stimulated with IL‐1β. Figure 5C indicates that IL‐1β increased FLS viability, while silencing of *Lrrc15* led to a suppression of FLS viability compared to that of the IL‐1β + siNC group. Consistent with the results of CCK‐8 assays, EdU staining demonstrated that IL‐1β promoted FLS proliferation, but silencing of *Lrrc15* inhibited cell proliferation induced by IL‐1β (Figure 5D). Additionally, it was observed that the migrated number of FLSs was increased compared to that of the control group, but silencing of siLrrc15 reduced the migrated number of FLSs in IL‐1β group (Figure 5E).

### 3.6 Silencing of *Lrrc15* Attenuates IL‐1β‐Induced Pro‐Inflammatory Cytokine Levels in FLS

**Figure 6:**
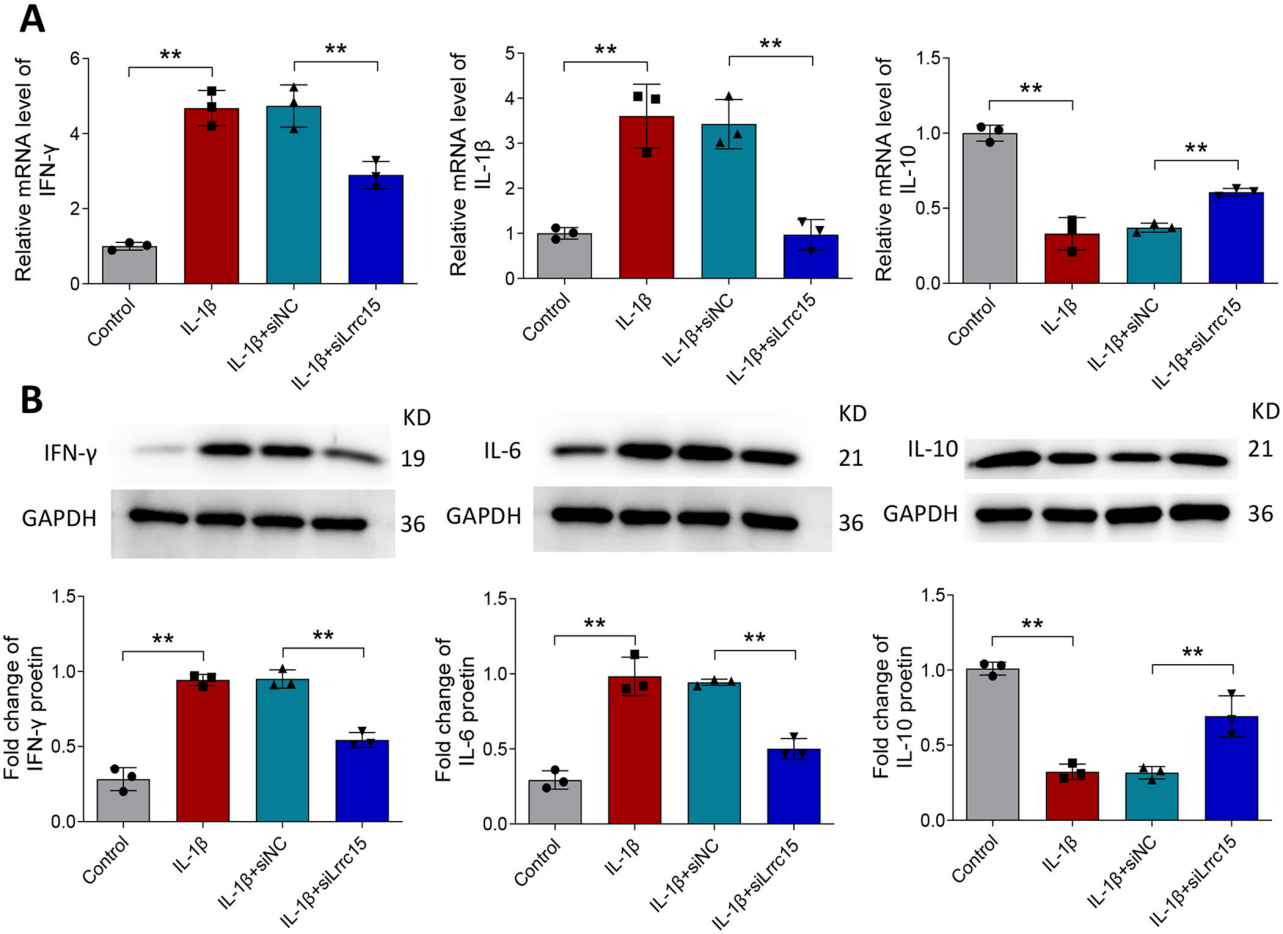
Effects of *Lrrc15* on IL‐1β‐induced pro‐inflammatory cytokine levels in FLSs in vitro. (A–B) Primary rat FLSs transfected with siNC or siLrrc15 were treated with 10 ng/mL IL‐1β for 24 h. The levels of IFN‐γ, IL‐6, and IL‐10 was measured by RT‐qPCR (A) and western blot (B). Data were presented as Mean ± SD and analyzed using the one‐way ANOVA. *N* = 3. **p < 0.01.

The changes of the inflammatory cytokines IFN‐γ, IL‐6, and IL‐10 in FLSs stimulated with IL‐1β were detected using RT‐qPCR and western blot analysis. Figure 6A indicates that IL‐1β stimulation increased the mRNA levels of *Ifng* and *Il6* and decreased the mRNA levels of *Il10*, while silencing of *Lrrc15* reversed the changes induced by IL‐1β. Moreover, the results of western blot also confirmed this (Figure 6B).

### 3.7 Silencing of *Lrrc15* Induces the Inhibition NF‐κB Signaling Pathway in FLS Stimulated With IL‐1β

**Figure 7:**
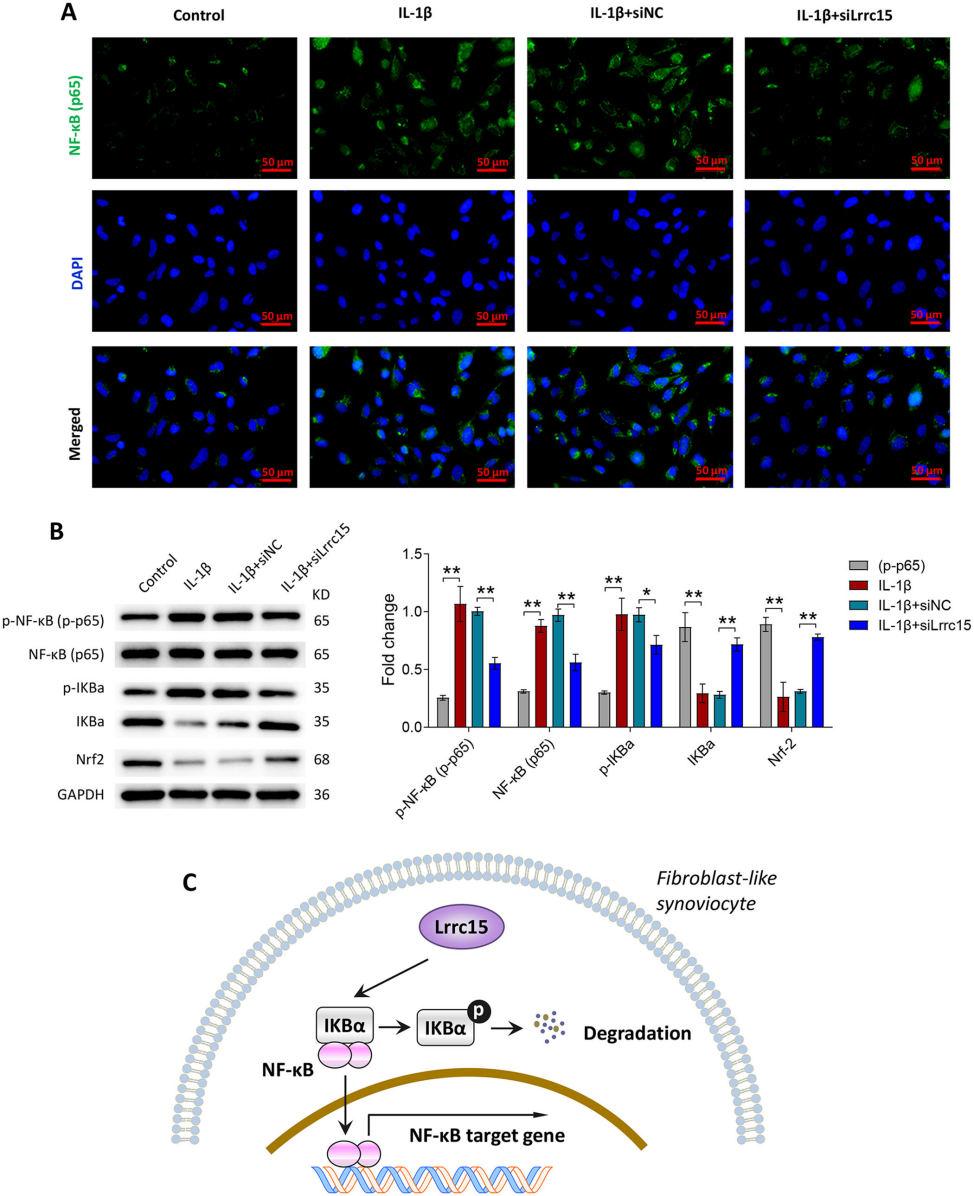
Regulation of *Lrrc15* on the NF‐κB signaling pathway in FLS in vitro. (A and B) Primary rat FLSs transfected with siNC or siLrrc15 were treated with 10 ng/mL IL‐1β for 24 h. NF‐κB (p65)‐positive cells were measured by immunofluorescence assay (A). The protein levels of NF‐κB (p65), p‐NF‐κB (p‐p65), p‐IKBα, IKBα, and Nrf2 were measured by western blot (B). (C) A schematic diagram displaying the regulatory relationship between *Lrrc15* and NF‐κB signaling pathway in FLS. Data were presented as Mean ± SD and analyzed using the one‐way ANOVA. *N* = 3. *p < 0.05; **p < 0.01.

Immunofluorescence assays demonstrated that IL‐1β induced greater NF‐κB (p65) nuclear transfer compared to that of the control group, while silencing of *Lrrc15* reduced the translocation of NF‐κB (p65) to the nucleus (Figure 7A). Further, Figure 7B revealed that IL‐1β promoted the protein expression of NF‐κB (p65), p‐NF‐κB (p‐p65), and p‐IKBα and inhibited the protein expression of IKBα and Nrf2, but silencing of *Lrrc15* reversed these changes of protein expression induced by IL‐1β. It seems that, the highly expressed *Lrrc15* in FLSs leads to IκBα degradation to induce NF‐κB activation and the expression of inflammatory cytokines (Figure 7C).

## 4 Discussion

Patients with RA typically exhibit joint swelling and stiffness that can lead to severe disability and can seriously affect their physical and mental quality of life [18]. At present, the pathogenesis of RA remains unclear. A better understanding of gene‐based mechanisms may help to clarify the pathogenesis of RA and provide potential approaches for treating or even preventing RA. In the current study, we revealed that *Lrrc15* may be a key regulator of RA pathogenesis. We found that LRRC15 was highly expressed in RA cells and animal models. The knockdown of *Lrrc15* inhibited NF‐κB pathway to control FLS proliferation, migration, and inflammatory response, and this finally limited the progression of RA.

The high‐level expression of *Lrrc15* in RA has recently been reported. Based on bioinformatics analysis, He et al. reported that *Lrrc15* was highly expressed in the synovial tissues of patients with RA, and its expression was positively correlated with the number of follicular helper T cells and M1 macrophages, while it was negatively correlated with the number of monocytes and resting dendritic cells [19]. By analyzing six RA synovial microarray data sets from the GEO database, Li et al. also confirmed the higher expression of *Lrrc15* in RA synovial tissues compared to that in normal synovial tissues [20]. Although *Lrrc15* did not exhibit a satisfactory diagnostic ability in the validation data set, *Lrrc15* was identified as a key gene involved in the dysregulation of arthritic microenvironment homeostasis [20]. Consistent with these previous findings, we observed high‐level expression of LRRC15 in the IL‐1β‐treated FLSs and synovial tissues of CIA rats, and this indicates that *Lrrc15* may participate in the development of RA.

In RA, decreased arthritis scores are related to reduced swelling, synovial hyperplasia, and synovial inflammatory cell infiltration [21]. Our data demonstrated that the knockdown of *Lrrc15* alleviated cartilage damage, inflammatory cell infiltration, synovial hyperplasia, and cartilage matrix degradation in CIA rats, indicating that knockdown of *Lrrc15* could alleviate disease progression in RA animal models. In the pathogenesis of RA, FLSs are the main effector cells in joint injury induced by RA [22]. FLSs of RA exhibit an aggressive phenotype, promoting proliferation and invasion, and they can produce excessive pro‐inflammatory cytokines and matrix‐degrading enzymes that lead to dysfunction and destruction of joints [23, 24]. In this study, we found that the knockdown of *Lrrc15* inhibited FLS proliferation, migration, and inflammatory responses. Consistent with our results, Ding et al. reported that *Lrrc15* overexpression in FLS increased cell proliferation, migration, invasion, and the release of pro‐inflammatory cytokines [25], suggesting the involvement of *Lrrc15* in regulating FLS activation and synovial inflammation.

In active synovial tissues, pro‐inflammatory cytokines are excessively produced and released to promote autoimmune inflammation and tissue damage [26]. In the pathogenesis and progression of RA, pro‐inflammatory cytokines, including TNF‐α, IFN‐γ, IL‐6, and IL‐1β, are key functional molecules [27]. Reducing the levels of inflammatory cytokines in FLSs have shown potentials in ameliorating the progression of RA [28, 29, 30]. Dayer reported that cell proliferation and the production of matrix metalloproteinases in chondrocytes and synovial cells could be induced by IL‐1β, and IL‐1β promoted cartilage degradation [31]. It is widely accepted that glucocorticoids can be utilized to treat RA, as they are effective in reducing the levels of pro‐inflammatory cytokines such as IL‐1β, TNF‐α, and IL‐6, while increasing the levels of the anti‐inflammatory cytokine IL‐10 [32, 33]. In this study, the knockdown of *Lrrc15* showed anti‐inflammatory functions in the IL‐1β‐treated FLSs and synovial tissues of CIA rats, and this indicated that the knockdown of *Lrrc15* could alleviate inflammatory response in RA.

A large number of studies have revealed that NF‐κB signaling pathway plays an important role in the pathogenesis and etiology of RA and takes part in regulating systemic inflammation, matrix degeneration, synovial hyperplasia, and bone loss [34, 35]. When NF‐κB signaling pathway is activated by various stimuli, IκBα is degraded, and this subsequently leads to NF‐κB (p65) transfer from cytoplasm to the nucleus [36]. After activation of the NF‐κB signaling pathway, various pro‐inflammatory genes are expressed that induce inflammation and aggravate RA progression [34, 35]. As an antioxidant protein, Nrf2 is a critical regulator of protective cellular processes [37]. There is functional crosstalk between Nrf2 and NF‐κB (p65), and the absence of Nrf2 leads to NF‐κB signaling pathway activation [38]. Nrf2 has been reported to inhibit RA progression by inhibiting the JAK‐STAT, NF‐κB, and MAPK signaling pathways [39]. In this study, the knockdown of *Lrrc15* inhibited NF‐κB signaling pathway and promoted the protein expression of Nrf2. Specifically, the knockdown of *Lrrc15* inhibited the inflammatory response by inhibiting the activation of NF‐κB signaling pathway in RA.

The consensus of researchers is that the pathogenesis of RA involves multiple genetic factors [40]. For instance, *Smoc2* [41], *Six1* [42], and *Alkbh5* [43], have been implicated with the disease's inflammation. Here, we for the first time demonstrated that high expression of LRRC15 is involved in the FLS activation and synovial inflammation in RA. Understanding the interplay between these factors not only contributes to a deeper insight into the mechanisms of the disease, but also provide possible candidate genes for the targeted treatment. Regardless, there are several limitations of the present paper and much work remains to be done in the following. Studies clarifying the association between *Lrrc15* expression and the disease onset, especially in the clinical context, as well as its function in other cell types, including B cells or monocytes are needed. The vast, and yet unexploited signaling pathways may participate in the role of *Lrrc15* in RA, further work is anticipated to reveal the regulation of *Lrrc15* on the downstream pathways. In addition, although recent papers have focused on investigating *Lrrc15* as it plays significant roles in the regulation of cell‐cell and cell‐matrix interactions [9], its physiological role is still largely unknown. The normal physiological role of *Lrrc15* should be further revealed to enrich our understanding of this gene.

In RA, the knockdown of *Lrrc15* inhibited the proliferation and migration of FLSs and the inflammatory response, and this might be related to the inhibition of the NF‐κB pathway. This study suggests *Lrrc15* as a new potential target for the treatment of RA.

## Author Contributions

**Miaomiao Xin:** conceptualization and data curation. **Guangtao Xia:** conceptualization and writing – original draft. **Xin Guan:** data curation and formal analysis. **Guangmin Xi:** data curation and formal analysis. **Min Fu:** conceptualization, data curation, and formal analysis. All authors reviewed the manuscript.

## Ethics Statement

The experimental protocol of our study was performed in accordance with the Guide for the Care and Use of Laboratory Animals and the experimental protocol approved by the Shandong Provincial Hospital Affiliated to Shandong First Medical University (Shandong Provincial Hospital).

## Consent

The authors have nothing to report.

## Conflicts of Interest

The authors declare no conflicts of interest.

## Data Availability

The data sets used and analyzed during the current study are available from the corresponding author on reasonable request.
